# Evaluation of the stiffness of the transplanted liver and spleen in recipients after liver transplantation on shear wave elastography

**DOI:** 10.1186/s12938-025-01481-7

**Published:** 2025-12-30

**Authors:** Jiaqi Li, Xi Yang, Mei Zhang, Le Ma, Xu Wang, Xinyu Wang, Xin Mao, Guangsen Li

**Affiliations:** Department of Ultrasound, the Second Affiliated Hospital of Dalian Medical University, Dalian, 116027 Liaoning China

**Keywords:** Shear wave elastography, Liver transplantation, Liver stiffness, Spleen stiffness, Transplanted liver fibrosis

## Abstract

**Background:**

Liver fibrosis after liver transplantation seriously affects graft survival. This study aimed to use shear wave elastography (SWE) to non-invasively evaluate the stiffness of the transplanted liver and spleen in liver transplant recipients.

**Methods:**

Seventy-one patients who were regularly reviewed in our hospital after liver transplantation (> 2 years) were enrolled and were divided into two groups: Group B (30 cases without splenomegaly before liver transplantation) and Group C (41 cases with splenomegaly before liver transplantation). Besides, we selected 35 normal controls as Group A. All patients underwent conventional ultrasound, SWE, and serum liver fibrosis markers exams to obtain the oblique diameter of right lobe of liver (ODRL), portal vein diameter (PVD), portal vein velocity (PVV), peak systolic velocity (PSV), splenic vein diameter (SVD), splenic length, splenic thickness, liver stiffness measurement (LSM), spleen stiffness measurement (SSM) and laboratory test results. Research the correlation between SWE parameters and serum liver fibrosis markers by Pearson linear analysis.

**Results:**

There was no difference in ODRL, PVD, PVV, and PSV among the three groups (all *P* > 0.05). Compared with Group A, hyaluronic acid (HA), laminin (LN), type III N-peptide collagen (PIIIP N-P), collagen type IV (IVC), and LSM were increased in Groups B and C (all* P* < 0.05) and were all higher in Group C than in Group B (all *P* < 0.05). Compared with Groups A and B, Group C was significantly higher in SVD, splenic length, splenic thickness, and SSM (all *P* < 0.05), whereas there was no difference in comparison of Group B with Group A (all *P* > 0.05). Pearson’s correlation analysis showed that LSM and HA, LN, PIIIN-P, and IVC were positively correlated.

**Conclusion:**

SWE is valuable to evaluate the stiffness of transplanted liver and spleen in recipients after liver transplantation, and preoperative splenomegaly is a factor influencing graft fibrosis after transplantation.

## Introduction

Chronic liver disease is a serious international public health problem, and liver fibrosis is a common feature of its progression, which can progress to cirrhosis or even end stage liver disease [1, 2]. For end stage liver disease such as primary hepatocellular carcinoma, decompensated liver cirrhosis, hepatic failure, and so on, liver transplantation is the most effective therapy to prolong patients’ lives and improve their life quality up to now [3]. Postoperative complications of liver transplantation are the crucial cause of postoperative mortality in recipients. The recipients are followed up for a long time in order to timely detect and accurately diagnose various complications, which is an important prerequisite for ensuring patients’ long-term survival. In the early years of liver transplantation, the surgical technique and the postoperative bleeding were key problems. With the development of medical technology and the systematic improvement of perioperative management, serious complications such as cellular rejection, anastomotic strictures and leaks, vascular complications, and recurrent primary liver tumors reduce graft survival rates [4, 5]. Nowadays, the clinical use of immunosuppressive drugs and the early detection of the disease by imaging have reduced the incidence of these complications, but it was shown that graft fibrosis is still prevalent in the long-term survival prognosis of the patients [6]. Graft fibrosis can affect patients’ quality of life as well as the survival time of the graft and eventually lead to even graft loss with its development, which increases patients’ mortality [7, 8]. Identifying recipients at risk for developing graft fibrosis and halting its progression are critical to reducing their mortality and avoiding retransplantation. Equally, finding tools for detecting and monitoring liver fibrosis is essential to improve recipients’ prognosis.

Shear wave elastography (SWE), as a quantitative and non-invasive assessment of estimating organ elasticity with good repeatability and accuracy, obtained organ stiffness by examining the propagation velocity of shear waves in tissue measured, which can detect the stiffness changes in the tissue before anatomical changes in the organ parenchyma have occurred [9, 10]. When liver fibrosis occurs, liver stiffness increases and liver elasticity decreases. And the liver is easily examined by ultrasound, which can be used to detect liver stiffness by SWE. In this study, we applied SWE to measure the stiffness of the grafted liver and spleen at steady state after liver transplantation in patients with or without splenomegaly before liver transplantation and analyzed the correlation between liver stiffness measurements and serological markers of hepatic fibrosis in order to assess whether the grafted liver of the recipients had developed fibrosis and the degree of progression of the fibrosis after transplantation with the aim of quantitatively and non-invasively providing a reference for the clinical diagnosis and treatment.

## Results

### General clinical parameters

The results of age, gender, BMI, SBP, DBP, and HR had no significant statistical difference for all subjects (all *P* > 0.05). (Table 1).

**Table 1:** Comparison of general clinical parameters among the three groups $(\overline{x}  \pm s)$

Parameters	Group A(n = 35)	Group B(n = 30)	Group C(n = 41)
Age (years)	60 (47, 64)	57 (45, 63)	53 (47, 61)
Gender (M/F)	30/5	27/3	34/7
BMI(kg/m^2^)	24.1 ± 2.5	23.2 ± 2.4	23.3 ± 1.8
SBP(mmHg)	124.3 ± 8.6	127.3 ± 11.2	123.1 ± 9.1
DBP(mmHg)	76.9 ± 5.3	77.3 ± 4.8	77.8 ± 5.4
HR(bpm)	78.4 ± 5.3	77.0 ± 6.1	78.2 ± 4.9

*M* male, *F* female, *BMI* body mass index, *SBP* systolic blood pressure, *DBP* diastolic blood pressure, *HR* heart rate, Group A: normal controls, Group B: patients without splenomegaly before liver transplantation, Group C: patients with splenomegaly before liver transplantation

### Conventional ultrasound parameters

There was no significant difference in ODRL, PVD, PVV, and PSV among the three groups (all* P* > 0.05). We found SVD, splenic length, and splenic thickness were significantly larger in Group C than in Group A and Group B (all *P* < 0.05), but there was no significant difference between Group B and Group A(all* P* > 0.05). (Table 2).

**Table 2:** Comparison of conventional ultrasound parameters among the three groups $(\overline{x}  \pm s)$

Parameters	Group A(n = 35)	Group B(n = 30)	Group C(n = 41)
ODRL (cm)	13.48 ± 1.34	14.06 ± 1.02	13.86 ± 1.18
PVD (cm)	0.88 ± 0.10	0.91 ± 0.09	0.92 ± 0.12
PVV (cm/s)	35.52 ± 11.15	36.67 ± 12.16	40.08 ± 10.61
PSV (cm/s)	52.16 ± 11.91	54.57 ± 13.62	58.17 ± 14.56
SVD (cm)	0.71 ± 0.16	0.78 ± 0.15	1.14 ± 0.23^*#^
Splenic length(cm)	10.24 ± 1.43	10.63 ± 1.62	15.08 ± 2.17^*#^
Splenic thickness (cm)	3.36 ± 0.41	3.52 ± 0.38	5.27 ± 0.71^*#^

*ODRL* oblique diameter of right lobe of liver, *PVD* portal vein diameter, *PVV* portal vein velocity, *PSV* peak systolic velocity, *SVD* splenic vein diameter

^*^P < 0.05 versus Group A

^#^*P* < 0.05 versus Group B

### SWE parameters

**Fig. 1:**
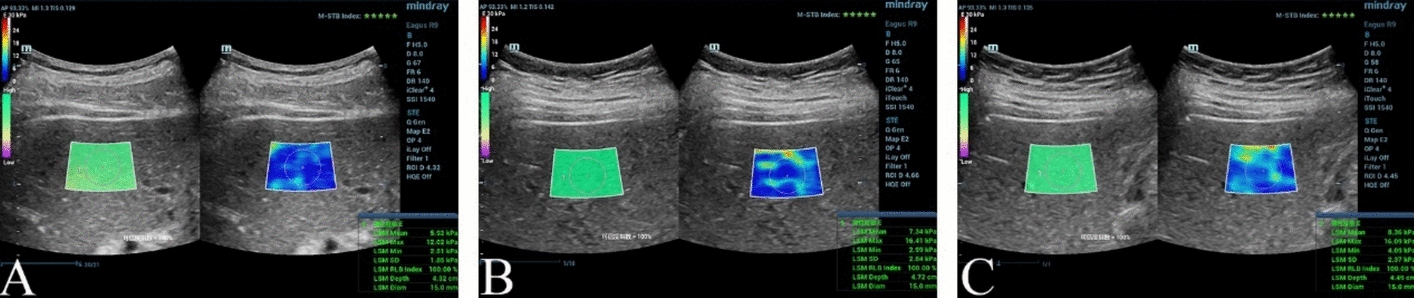
A LSM in Group A (LSM 5.92 kPa), B LSM in Group B (LSM 7.34 kPa), LSM in C Group C (LSM 8.36 kPa)

**Fig. 2:**
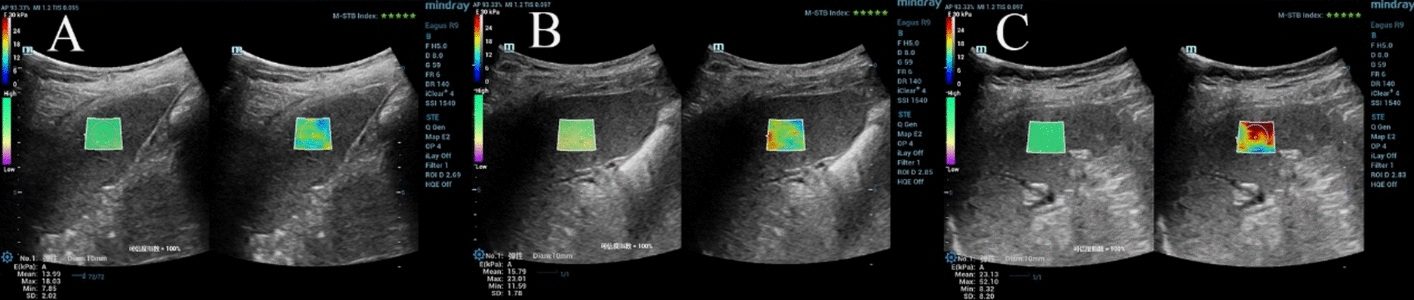
A SSM in Group A (SSM 13.99 kPa), B SSM in Group B (SSM 15.79 kPa),C SSM in Group C (SSM 23.13 kPa)

Compared with Group A, the LSM was increased in Groups B and C (*P* < 0.05), and was higher in Group C than in Group B (*P* < 0.05). For the SSM, the Group C was higher than Group A and Group B, but there was no statistical difference between Groups A and B (*P* > 0.05). (Table 3 and Fig. 1 and 2).

**Table 3:** Comparison of LSM and SSM among the three groups $(\overline{x}  \pm s)$

Parameters	Group A(n = 35)	Group B(n = 30)	Group C(n = 41)
LSM (kPa)	5.41 ± 0.82	6.73 ± 0. 96^*^	7.51 ± 1.03^*#^
SSM (kPa)	13.81 ± 1.94	15.06 ± 2.35	23.91 ± 3.14^*#^

*LSM* liver stiffness measurement, *SSM* spleen stiffness measurement

^*^P < 0.05 versus Group A

^#^*P* < 0.05 versus Group B

### Serum liver fibrosis markers

Compared with Group A, HA, LN, PIIIP N-P, and IVC were increased in Groups B and C(all* P* < 0.05) and were at a higher level in Group C than in Group B (all *P* < 0.05). (Table 4).

**Table 4:** Comparison of serum liver fibrosis markers among the three groups $(\overline{x}  \pm s)$

Parameters	Group A(n = 35)	Group B(n = 30)	Group C(n = 41)
HA(ng/ml)	42.57 ± 10.38	86.85 ± 13.87^*^	118.13 ± 16.98^*#^
LN(ng/ml)	25.62 ± 2.59	31.55 ± 2.41^*^	35.58 ± 2.84^*#^
PIIIP N-P(ng/ml)	8.61 ± 0.84	19.23 ± 0.93^*^	21.62 ± 1.23^*#^
IVC(ng/ml)	23.07 ± 2.81	46.78 ± 3.62^*^	61.34 ± 4.81^*#^

*HA* hyaluronic acid, *LN* laminin, *PIIIP N-P* type III N-peptide collagen, *IVC* collagen type IV

^*^P < 0.05 versus Group A

^#^*P* < 0.05 versus Group B

### Correlation analysis

**Fig. 3:**
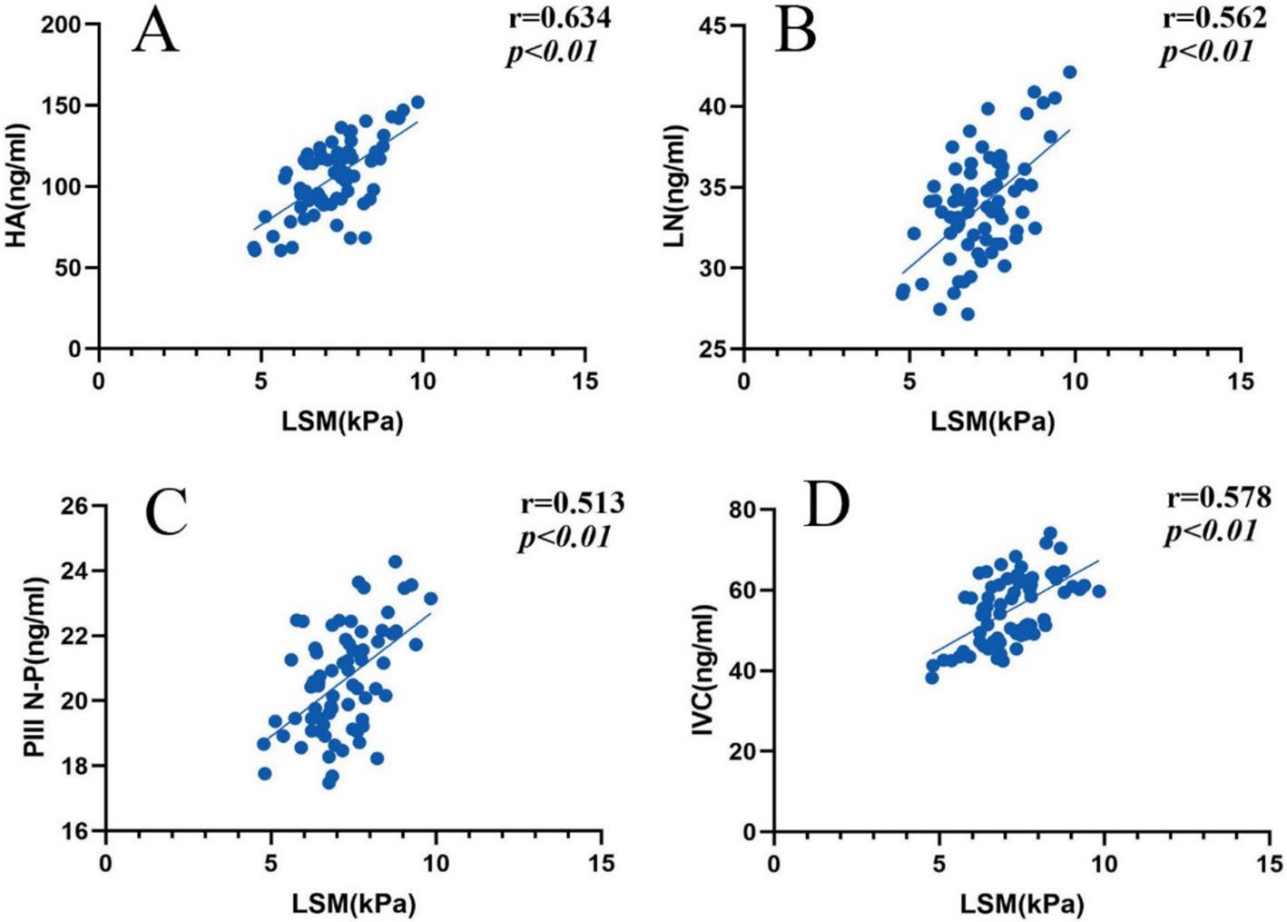
Correlation analysis scatter plot. *LSM* liver stiffness measurement, *HA* hyaluronic acid, *LN* laminin, *PIIIP N-P* type III N-peptide collagen, *IVC* collagen type IV

Pearson correlation analysis showed that LSM was positively correlated with HA, LN, PIIIP N-P, and IVC (*r* = 0.634, 0.562, 0.513, 0.578, all *P* < 0.01). (Fig. 3).

### Repeatability test

Both LSM and SSM showed satisfactory intra-observer and inter-observer repeatability (ICCs = 0.897, 0.864, 0.918, and 0.873). (Table 5).

**Table 5:** Reliability analysis of LSM and SSM

	Intra-observer			Inter-observer		
Parameters	ICC	95% consistency limit	P	ICC	95% consistency limit	P
LSM	0.897	0.734–0.957	< 0.001	0.864	0.632–0.945	< 0.001
SSM	0.918	0.651–0.972	< 0.001	0.873	0.714–0.943	< 0.001

*SWE* shear wave elastography, *LSM* liver stiffness measurement, *SSM* spleen stiffness measurement, *ICC* intra-observer correlation coefficients

## Discussion

Liver fibrosis, as a gradually developing pathological process, is characterized by a gradual buildup of extracellular matrix (ECM) produced by fibrogenic myofibroblasts [11, 12], which damages the physiological structure of the liver. And as the disease progresses, it affects the patient more. Early detection and therapeutic intervention can slow the progression of the disease or even reverse it [13]. However, when liver fibrosis progresses to the stage of cirrhosis and end-stage liver disease, it can only be cured by liver transplantation. While the ischemia–reperfusion process of blocking blood flow and subsequent restoration of blood flow during liver transplantation can cause hemodynamic abnormalities in the grafted liver of the recipients in the early or even mid-postoperative period, with gradual recovery of the patient over time. There was no difference in ODRL, PVD, PVV, and PSV of all the participants in this study, indicating that the corresponding hemodynamic fluctuations in the patient's transplanted liver have stabilized [8, 14].

SWE is conducted to follow the propagation of the shear wave and measure its velocity, which is directly related to tissue stiffness. The harder the tissues, the faster the shear wave propagates. [15]. SWE, as a convenient, accurate, and reproducibly noninvasive means of assessing organ elasticity, is now widely used in the evaluation of the stiffness of superficial organ lesions such as the thyroid and breast [16, 17]. Previous studies had shown that SWE can be used for measuring liver stiffness in viral hepatitis and fatty liver disease to identify the progress of liver fibrosis [18, 19]. In other literature, SWE had been shown to be satisfactory for evaluating splenic stiffness to predict hepatic dysfunction and esophageal varices in patients with cirrhosis [20, 21]. In recent years, several experts have used it for the routine follow-up of postoperative liver transplantation patients, which can avoid invasive biopsy examination of the patients by performing elastography of the grafted liver to predict the occurrence of complications and assess the function and prognosis of the transplanted liver [22, 23].

The portal vein is formed by the confluence of the splenic and superior mesenteric veins. Before transplantation, the blood flow back caused by portal hypertension led to splenic and superior mesenteric veins congestion hypertension. When the reflux of the spleen vein is blocked and exceeds the spleen's compensation ability, the spleen will have spleen sinusoid expansion, the hyperactivation of spleen pulp tissue, congestion splenomegaly, and spleen sclerosis, which then results in increased spleen stiffness and decreased spleen elasticity [24, 25]. In this study, the splenic length, splenic thickness, SVD, and SSM were higher in Group C than in Groups B and A (all* P* < 0.05), while there was no significant difference between Group B and Group A (all *P* > 0.05), suggesting that the patient's preoperative splenomegaly and spleen fibrosis had not recovered. The reason may be that the changes of spleen stiffness during splenomegaly depend on splenic congestion and fibrosis [26]. Although liver transplantation relieves the patients’ preoperative high intrahepatic resistance, the extrahepatic high circulation state will still exist for a period of time, which causes increased splenic vein blood flow and congestion splenomegaly. So, the preoperative splenomegaly and spleen fibrosis caused by portal hypertension will still exist and even be irreversible after liver transplantation [14, 27, 28].

In this study, LSM was increased in Groups B and C compared to Group A (*P* < 0.05), and it was higher in Group C than in Group B (*P* < 0.05). This indicates that patients who underwent liver transplantation are more likely to develop liver fibrosis after liver transplantation and also suggests that preoperative splenomegaly is also an influential factor in postoperative grafted liver fibrosis, which can further lead to increased liver stiffness. On the one hand, the reason for the increase in liver stiffness may be due to liver transplantation. The grafted liver underwent an ischemia–reperfusion process through liver transplantation, which could damage ischemia-sensitive biliary epithelial cells. This in turn causes intrahepatic cholestasis, and cholestasis combined with bile duct injury leads to the occurrence of hepatic fibrosis [29]. It may also be that ischemia–reperfusion causes an increase in the expression of pro-fibrotic genes and the infiltration of myofibroblasts, which results in an increase in collagen deposition, ultimately leading to an increase in liver stiffness [30]. In addition to this, it is considered that patients taking immunosuppressive drugs postoperatively increase the incidence of liver fibrosis-related diseases, thus causing increased liver stiffness [31]. On the other hand, the reason for the increase in liver stiffness may be due to splenomegaly. In patients with preoperative splenomegaly, the portal vein blood flow briefly increased and the liver cells significantly regenerated after liver transplantation due to the release of high intrahepatic resistance and the existence of extrahepatic hyperdynamic circulation, which resulted in increased liver stiffness. It may also be that splenic-derived immune cells in patients with splenomegaly exacerbate hepatic fibrosis through the spleen-liver axis [32], further increasing liver stiffness.

Direct serological markers of liver fibrosis are biomarkers that directly target the extracellular matrix scarring component that causes the development of liver fibrosis, and their levels in serum gradually increase with the exacerbation of the degree of liver fibrosis and decrease with treatment [33, 34]. The liver fibrosis biomarkers, including HA, LN, PIIIP N-P, and IVC are direct serologic liver fibrosis markers that are commonly used for monitoring the change in the extent of liver fibrosis clinically. In this study, the liver fibrosis biomarkers were increased in liver transplantation recipients compared with Group A (all *P* < 0.05), and were higher in Group C than in Group B ( all *P* < 0.05), suggesting that fibrosis was more prone to occur in the grafted liver of recipients after liver transplantation and was to a higher extent in patients with splenomegaly before liver transplantation than without splenomegaly. Correlation analysis research revealed a positive correlation between the liver fibrosis biomarkers and LSM in liver transplantation recipients, suggesting that the liver fibrosis biomarkers also increased with the increase of LSM, and concluded that SWE is valuable in assessing liver fibrosis.

## Limitations

There are some limitations in this study. Firstly, it was performed at a single institution within a limited period, and the sample size is not yet sufficient, which was not representative of the Chinese population. Secondly, this study lacks liver and spleen biopsies for comparison, so it cannot reflect the comprehensive histological information provided by liver and spleen biopsies. Finally, LSM and SSM measurements are inevitably slightly influenced by operator subjectivity. These limitations underscore the need for further standardization and research to optimize the clinical utility of SWE in liver transplantation patients. Going forward, we will get further research to study the postoperative detection value of SWE for liver transplantation patients by large-scale, prospective, multicenter data and combining SWE results with other clinical parameters.

## Conclusion

In summary, SWE can be applied to assess the grafted liver stiffness and spleen stiffness in recipients after liver transplantation. And the extent and progression of postoperative graft fibrosis is more rapid in patients with pre-transplantation splenomegaly than in patients without splenomegaly, which shows that splenomegaly before liver transplantation is the influencing factor of postoperative grafted liver fibrosis.

## Methods

### Study subjects

Seventy-one patients were enrolled who met the following conditions, including stable health status and after liver transplantation (> 2 years) with routine follow-up from June 2022 to June 2024 in our hospital. All the recipients were rated according to the Model for End-Stage Liver Disease (MELD) score before liver transplantation, and the results were as follows, which met liver transplantation standards. (Table 6).

**Table 6:** MELD score of all the recipients

Scores	Sense	Numbers
< 12		0
12–18	Be placed on the waiting list for liver transplantation	21
18–25	Require liver transplantation	49
25–30	Require emergency liver transplantation	1
> 30	Require urgent liver transplantation for rescue treatment	0

Exclusion criteria: ① secondary hepatocellular carcinoma ② post-transplant complications, including graft immune rejection, hepatic artery thrombosis, hepatic artery stenosis, choledochal anastomotic stenosis, and cholestasis ③ non-first liver transplantation ④ postoperative recurrence of hepatitis or hepatocellular carcinoma ⑤ steatosis of the liver graft ⑥ hyperglycemic state ⑦ splenectomy, and so on.

71 patients were divided into two groups: Group B (30 cases without splenomegaly before liver transplantation, M:F = 27:3, median age 57, range 37–71 years) and Group C (41 patients with splenomegaly before liver transplantation, M:F = 34:7, median age 53, range 38–72 years). We follow the Chinese ultrasound guidelines to define splenomegaly as the spleen length diameter being larger than 12 cm and the spleen thick diameter being larger than 4 cm by ultrasound. While 35 healthy controls were selected as Group A (M:F = 30:5, median age 60, range 35–83 years). Of Group B, preoperative primary diseases included 12 cases of viral hepatitis combined with hepatocellular carcinoma, 6 cases of cirrhosis combined with hepatocellular carcinoma, 5 cases of simple hepatocellular carcinoma, 1 case of hepatocellular carcinosarcoma, 2 cases of polycystic liver, 2 cases of acute liver failure, 1 case of severe hepatitis, and 1 case of cholangiocellular carcinoma. And the preoperative primary diseases of patients in Group C were cirrhosis combined with hepatocellular carcinoma (25 cases) and simple cirrhosis (16 cases).

All participants were given written informed consent, and the study was approved by the Ethics Committee of the Second Affiliated Hospital of Dalian Medical University (KY2024-146–02). The liver donations in this article all were deceased whole livers from adults, which came from patients who died unexpectedly and those in the intensive care unit of our hospital, as well as death row inmates who were willing to contribute for society. All donors were voluntary, and their families had signed the relevant agreements. The transplantation procedures were all piggyback liver transplantations, which involved the donor's inferior vena cava anastomosed to the recipient's hepatic vein, preserving the recipient's retrohepatic inferior vena cava, as well as anastomosis of the portal vein, the hepatic artery, and the biliary duct between donor and recipient.

### General clinical and laboratory parameters

The age, gender, systolic blood pressure (SBP), diastolic blood pressure (DBP), heart rate (HR), height, and weight of all participants were recorded, and body mass index (BMI) was calculated. The liver fibrosis biomarkers were collected, including hyaluronic acid (HA), serum laminin (LN), type III procollagen (PIIIP N-P), and type IV collagen (IVC).

### Conventional ultrasound

The ultrasound instrument Mindray Eagus R9, equipped with SWE software, was performed, and the probe of SC6-1 with a frequency range of 1.0 to 5.0 MHz was selected. The subjects were in a supine position on the examination bed and ensured to have fasted for 6–8 h and rested in a quiet environment for at least 20 min. Conventional ultrasound was applied for obtaining the oblique diameter of the right lobe of the liver (ODRL), portal vein diameter (PVD), portal vein velocity (PVV), peak hepatic artery velocity (PSV), splenic length, splenic thickness, and splenic vein diameter (SVD).

### SWE

Switch the mode to shear wave elastography (SWE) and adjust the image depth to 8 cm to measure liver and spleen stiffness. Place the probe in the right intercostal space, and a sampling frame (40 mm × 30 mm) was placed in the liver parenchyma without intrahepatic ductal structures about 1-2 cm below the hepatic envelope. After that, patients were required to hold their breath, and liver stiffness measurement (LSM) recorded the region of interest (ROI) within a 2 cm diameter when the sampling frame image was color-filled and of reliable quality. While the probe was placed in the left intercostal area, we adjusted a sampling frame (15 mm × 15 mm) under the splenic envelope. Then spleen stiffness measurement (SSM) within the ROI (1 cm) was obtained with the same instrumental conditions and measurement method as for liver stiffness measurement.

During the whole procedure, the operator should pay attention to stabilizing the probe and communicate with the patient to obtain the patient’s breathing cooperation. All measured parameters were repeated five times to take the mean value. All the above ultrasound data measurements were completed by two ultrasound doctors with more than 5 years of work experience applying the same ultrasound instrument, the Mindray Eagus R9.

### Repeatability test

Twenty-five randomly selected subjects underwent LSM and SSM by two sonologists for inter-observer analysis. One week later, one sonologist repeated measurements on the same 25 subjects using the same machine and measurements for intra-observer assessment. Intra- and inter-observer variability were expressed as intraclass correlation coefficients (ICCs).

### Statistical analysis

Data processing, statistical analysis, and graph generation were performed using SPSS software (version 27.0) and GraphPad Prism (version 8.0). Normality of distribution was assessed using the Shapiro–Wilk test. Continuous variables following a normal distribution are presented as mean ± standard deviation **(__ ± sx)**. Comparisons among multiple groups were conducted using one-way analysis of variance (ANOVA) or the least significant difference *t-*test (LSD-*t* test). Non-normally distributed data were expressed as median and interquartile range [M (Q1, Q3)] and compared among groups using the Kruskal–Wallis test (K-W test). Categorical data are expressed as frequencies and percentages and were compared using the chi-square test. Correlations between the serum liver fibrosis markers (HA, LN, PIIIP N-P, and IVC) and LSM were analyzed using Pearson’s correlation coefficient. *P* < 0.05 was considered to be statistically significant.

## Data Availability

No datasets were generated or analysed during the current study.

## Abbreviations

SWE: Shear wave elastography
LSM: Liver stiffness measurement
SSM: Spleen stiffness measurement
ODRL: Oblique diameter of right lobe of liver
PVD: Portal vein diameter
PVV: Portal vein velocity
PSV: Peak systolic velocity
SVD: Splenic vein diameter
HA: Hyaluronic acid
LN: Laminin
PIIIP N-P: Type III N-peptide collagen
IVC: Collagen type IV
BMI: Body mass index
SBP: Systolic blood pressure
DBP: Diastolic blood pressure
HR: Heart rate

## References

[1] Berumen J, Baglieri J, Kisseleva T, Mekeel K. Liver fibrosis: pathophysiology and clinical implications. WIREs Mech Dis. 2021;13(1):e1499. 10.1002/wsbm.1499. 32713091 10.1002/wsbm.1499 PMC9479486

[2] Udompap P, Kim D, Kim WR. Current and future burden of chronic nonmalignant liver disease. Clin Gastroenterol Hepatol. 2015;13(12):2031–41. 10.1016/j.cgh.2015.08.015. 26291665 10.1016/j.cgh.2015.08.015 PMC4618163

[3] Bayramov N, Yilmaz S, Salahova S, Bashkiran A, Zeynalov N, Isazade E, et al. Liver graft and spleen elastography after living liver transplantation: our first results. Transplant Proc. 2019;51(7):2446–50. 10.1016/j.transproceed.2019.01.184. 31405739 10.1016/j.transproceed.2019.01.184

[4] Buros C, Dave AA, Furlan A. Immediate and late complications after liver transplantation. Radiol Clin North Am. 2023;61(5):785–95. 10.1016/j.rcl.2023.04.002. 37495287 10.1016/j.rcl.2023.04.002

[5] Craig EV, Heller MT. Complications of liver transplant. Abdom Radiol. 2021;46(1):43–67. 10.1007/s00261-019-02340-5. 10.1007/s00261-019-02340-5 31797026

[6] Jiang YZ, Zhao XY, Zhou GP, Wei L, Qu W, Zeng Z-G, et al. Impact of immunosuppression level on liver allograft fibrosis after pediatric liver transplantation: a retrospective cohort study. Int J Surg. 2023;109(11):3450–8. 10.1097/JS9.0000000000000631. 37578449 10.1097/JS9.0000000000000631 PMC10651304

[7] Navin PJ, Olson MC, Knudsen JM, Venkatesh SK. Elastography in the evaluation of liver allograft. Abdom Radiol. 2021;46(1):96–110. 10.1007/s00261-019-02400-w. 10.1007/s00261-019-02400-w PMC8497001 31950204

[8] Azhie A, Sharma D, Sheth P, et al. A deep learning framework for personalised dynamic diagnosis of graft fibrosis after liver transplantation: a retrospective, single Canadian centre, longitudinal study. Lancet Digit Health. 2023;5(7):e458. 37210229 10.1016/S2589-7500(23)00068-7

[9] Ferraioli G, Barr RG. Ultrasound liver elastography beyond liver fibrosis assessment. World J Gastroenterol. 2020;26(24):3413–20. 10.3748/wjg.v26.i24.3413. 32655265 10.3748/wjg.v26.i24.3413 PMC7327790

[10] Barr RG, Ferraioli G, Palmeri ML, Goodman ZD, Garcia-Tsao G, Rubin J, et al. Elastography assessment of liver fibrosis: Society of Radiologists in Ultrasound consensus conference statement. Radiology. 2015;276(3):845–61. 10.1148/radiol.2015150619. 26079489 10.1148/radiol.2015150619

[11] Zhang M, Serna-Salas S, Damba T, Borghesan M, Demaria M, Moshage H. Hepatic stellate cell senescence in liver fibrosis: characteristics, mechanisms and perspectives. Mech Ageing Dev. 2021;199:111572. 10.1016/j.mad.2021.111572. 34536446 10.1016/j.mad.2021.111572

[12] Roehlen N, Crouchet E, Baumert TF. Liver fibrosis: mechanistic concepts and therapeutic perspectives. Cells. 2020. 10.3390/cells9040875. 10.3390/cells9040875 PMC7226751 32260126

[13] Chen Z, Ma Y, Cai J, Sun M, Zeng L, Wu F, et al. Serum biomarkers for liver fibrosis. Clin Chim Acta. 2022;537:16–25. 10.1016/j.cca.2022.09.022. 36174721 10.1016/j.cca.2022.09.022

[14] Nam NH, Taura K, Yao S, Kaido T, Uemoto Y, Kimura Y, et al. Pretransplantation splenomegaly frequently persists after liver transplantation and can manifest as hypersplenism and graft fibrosis - a retrospective study. Transpl Int. 2020;33(12):1807–20. 10.1111/tri.13761. 33166011 10.1111/tri.13761

[15] Nakano M, Kuromatsu R, Kawaguchi T. Ultrasonographic assessment of tissue stiffness: recent progress in transient elastography and shear wave elastography in the liver and various organs. Kurume Med J. 2024;70(1.2):1–10. 38763738 10.2739/kurumemedj.MS7012010

[16] Barr RG, Engel A, Kim S, Tran P, De Silvestri A. Improved breast 2D SWE algorithm to eliminate false-negative cases. Invest Radiol. 2023;58(10):703–9. 10.1097/RLI.0000000000000972. 36939607 10.1097/RLI.0000000000000972

[17] Gao XQ, Ma Y, Peng XS, Wang L-L, Li H-X, Zheng X-L, et al. Diagnostic performance of C-TIRADS combined with SWE for the diagnosis of thyroid nodules. Front Endocrinol (Lausanne). 2022;13:939303. 10.3389/fendo.2022.939303. 36147579 10.3389/fendo.2022.939303 PMC9486702

[18] Song L, Zhao L, Deng J, Meng F, Wu X, Lu Q, et al. Staging liver fibrosis in patients with chronic hepatitis B using two-dimensional shear wave elastography based on histopathological findings: a prospective multicenter study. Quant Imaging Med Surg. 2023;13(4):2376–87. 10.21037/qims-22-831. 37064406 10.21037/qims-22-831 PMC10102744

[19] Karagiannakis DS, Markakis G, Lakiotaki D, Cholongitas E, Vlachogiannakos J, Papatheodoridis G. Comparing 2D-shear wave to transient elastography for the evaluation of liver fibrosis in nonalcoholic fatty liver disease. Eur J Gastroenterol Hepatol. 2022;34(9):961–6. 10.1097/MEG.0000000000002412. 35913779 10.1097/MEG.0000000000002412

[20] Karagiannakis DS, Voulgaris T, Markakis G, Lakiotaki D, Michailidou E, Cholongitas E, et al. Spleen stiffness can predict liver decompensation and survival in patients with cirrhosis. J Gastroenterol Hepatol. 2023;38(2):283–9. 10.1111/jgh.16057. 36346036 10.1111/jgh.16057

[21] Zhou H, Zhang Z, Zhang J, Sang L, Liu L, Gong X, et al. Performance of spleen stiffness measurement by 2D-shear wave elastography in evaluating the presence of high-risk varices: comparative analysis of idiopathic portal hypertension versus hepatitis B virus. BMC Med Imaging. 2023;23(1):30. 10.1186/s12880-023-00977-9. 36759764 10.1186/s12880-023-00977-9 PMC9909910

[22] Valente G, Rinaldi L, Moggio G, Piai G. Point shear wave elastography and vibration controlled transient elastography for estimating liver fibrosis in a cohort of liver transplant patients. Eur Rev Med Pharmacol Sci. 2020;24(13):7357–65. 10.26355/eurrev_202007_21903. 32706074 10.26355/eurrev_202007_21903

[23] Liu WY, Li XX, Fu XY, Wu X-D, Wang X, Guo Y, et al. Combination of liver graft sonographic grading and point shear wave elastography to reduce early allograft dysfunction after liver transplantation. Eur Radiol. 2020;30(9):5191–9. 10.1007/s00330-020-06842-5. 32328762 10.1007/s00330-020-06842-5

[24] Sharma P, Kirnake V, Tyagi P, Bansal N, Singla V, Kumar A, et al. Spleen stiffness in patients with cirrhosis in predicting esophageal varices. Am J Gastroenterol. 2013;108(7):1101–7. 10.1038/ajg.2013.119. 23629600 10.1038/ajg.2013.119

[25] Yoshida H, Shimizu T, Yoshioka M, Matsushita A, Kawano Y, Ueda J, et al. The role of the spleen in portal hypertension. J Nippon Med Sch. 2023;90(1):20–5. 10.1272/jnms.JNMS.2023_90-104. 36908126 10.1272/jnms.JNMS.2023_90-104

[26] Batur A, Alagoz S, Durmaz F, et al. Measurement of spleen stiffness by shear-wave elastography for prediction of splenomegaly etiology. Ultrasound Q. 2019;35(2):153–6. 30601437 10.1097/RUQ.0000000000000403

[27] Mitsuhashi Y, Wakiya T, Ishido K, Kudo D, Kimura N, Sato K, et al. Long-term changes in spleen volume after living donor liver transplantation in pediatric recipients. Transplant P. 2018;50(9):2723–5. 10.1016/j.transproceed.2018.04.028. 10.1016/j.transproceed.2018.04.028 30348453

[28] Iimuro Y, Yada A, Okada T, et al. Cytoglobin-expressing cells in the splenic cords contribute to splenic fibrosis in cirrhotic patients. Histol Histopathol. 2020;35(11):1319–28. 32945524 10.14670/HH-18-257

[29] Pinzani M, Luong TV. Pathogenesis of biliary fibrosis. Biochim Biophys Acta (BBA). 2018;1864(4 Pt B):1279–83. 10.1016/j.bbadis.2017.07.026 28754450

[30] Liu H, Man K. New insights in mechanisms and therapeutics for short- and long-term impacts of hepatic ischemia reperfusion injury post liver transplantation. Int J Mol Sci. 2021. 10.3390/ijms22158210. 10.3390/ijms22158210 PMC8348697 34360975

[31] Choudhary NS, Saigal S, Saraf N, Soin AS. Predictors of de novo nonalcoholic fatty liver disease after liver transplantation and associated fibrosis. Liver Transpl. 2019;25(6):967–8. 10.1002/lt.25450. 30884094 10.1002/lt.25450

[32] Han C, Zhai Y, Wang Y, Peng X, Zhang X, Dai B, et al. Intravital imaging of splenic classical monocytes modifying the hepatic CX3CR1(+) cells motility to exacerbate liver fibrosis via spleen-liver axis. Theranostics. 2024;14(5):2210–31. 10.7150/thno.87791. 38505603 10.7150/thno.87791 PMC10945343

[33] Karsdal MA, Daniels SJ, Holm Nielsen S, Bager C, Rasmussen DGK, Loomba R, et al. Collagen biology and non-invasive biomarkers of liver fibrosis. Liver Int. 2020;40(4):736–50. 10.1111/liv.14390. 31997561 10.1111/liv.14390

[34] Aleknavičiūtė-Valienė G, Banys V. Clinical importance of laboratory biomarkers in liver fibrosis. Biochem Med (Zagreb). 2022;32(3):030501. 10.11613/BM.2022.030501. 36277426 10.11613/BM.2022.030501 PMC9562801

